# Prognostic Value of the Red Cell Distribution Width in Patients with Sepsis-Induced Acute Respiratory Distress Syndrome: A Retrospective Cohort Study

**DOI:** 10.1155/2021/5543822

**Published:** 2021-06-02

**Authors:** Huabin Wang, Junbin Huang, Wenhua Liao, Jiannan Xu, Zhongyuan He, Yong Liu, Zhijie He, Chun Chen

**Affiliations:** ^1^Division of Hematology/Oncology, Department of Pediatrics, The Seventh Affiliated Hospital of Sun Yat-Sen University, Shenzhen 518107, China; ^2^Department of Pediatric Intensive Care Unit, The Seventh Affiliated Hospital of Sun Yat-Sen University, Shenzhen 518107, China; ^3^Department of Intensive Care Unit, Sun Yat-Sen Memorial Hospital of Sun Yat-Sen University, Guangzhou 510000, China; ^4^Center of Digestive Disease, The Seventh Affiliated Hospital of Sun Yat-Sen University, Shenzhen 518107, China; ^5^Department of Orthopedics, The Seventh Affiliated Hospital of Sun Yat-Sen University, Shenzhen 518107, China

## Abstract

**Objective:**

The prognostic value of the red cell distribution width (RDW) in patients with sepsis-induced acute respiratory distress syndrome (ARDS) is still elusive. This study is aimed at determining whether RDW is a prognostic indicator of sepsis-induced ARDS.

**Methods:**

This retrospective cohort study included 1161 patients with sepsis-induced ARDS. The datasets were acquired from the Medical Information Mart for Intensive Care III database. The locally weighted scatter-plot smoothing technique, Cox regression, Kaplan-Meier estimator, and subgroup analysis were carried out to evaluate the association between RDW and 90-day mortality.

**Results:**

The RDW and mortality had a roughly linear increasing relationship. The Cox regression model results were as follows: for level 2 (14.5% < RDW < 16.2%), hazard ratio (HR) = 1.35, 95% confidence interval (CI) = 1.03–1.77, and for level 3 (RDW ≥ 16.2%), HR = 2.07, 95% CI = 1.59–2.69. The following results were obtained when RDW was treated as a continuous variable: HR = 1.11, 95%CI = 1.06–1.15. The *P* values of the interaction between the RDW and covariates were greater than 0.05.

**Conclusion:**

RDW is a new independent prognostic marker for patients with sepsis-induced ARDS.

## 1. Introduction

Sepsis is caused by an imbalance in a host's response to infection and can lead to systemic multiple-organ dysfunction [1]. The lung is the first organ with the highest incidence rate of sepsis, and acute lung injury (ALI) is the main manifestation. ALI can further develop into acute respiratory distress syndrome (ARDS), an emergency and critical illness in the intensive care unit (ICU). It can cause excessive and uncontrolled inflammatory reactions [2], resulting in a clinical mortality rate (MR) as high as 35%–40% [3]. Therefore, early discrimination of high-risk sepsis-induced ARDS patients with worse prognoses is extremely important.

The red cell distribution width (RDW) is commonly assessed as part of a complete blood count and is often used to identify different types of anemia. RDW has received much attention from the healthcare community as a new diagnostic and prognostic indicator in recent years. Several studies have shown a close association between RDW and the prognosis of burns [4], pancreatitis [5], peritonitis [6], hepatitis B-related diseases [7], cardiovascular diseases [8, 9], and cancer [10–13]. However, no study has reported on the association between RDW and the prognosis of sepsis-induced ARDS patients. Moreover, clinical indicators for evaluating the prognosis of sepsis-induced ARDS patients are lacking. Therefore, this research is aimed at determining the predictive value of RDW in the MR of sepsis-induced ARDS patients.

## 2. Methods

### 2.1. Introduction to the Medical Database

The Medical Information Mart for Intensive Care III (MIMIC-III) V.1.4 database is a freely accessible critical care database that contains the clinical data of at least forty thousand critically ill patients hospitalized at the Beth Israel Deaconess Medical Center of Harvard Medical School between 2001 and 2012 (58,976 inpatients in total) [14]. The MIMIC-III database consists of comprehensive patient data such as biochemical, demographic, and physiological data as well as clinical diagnostics and medical treatment records. The MIMIC-III database not only has a large sample size and rich data types but also high-quality and high-reliability data. It is a treasure chest for clinical research in the field of critical care medicine. Wang obtained access to the database and was involved in data extraction (Certification No. 36132199).

### 2.2. Selection Criteria

We focused on the patients who were admitted to the ICUs from 2008 to 2012. All patients were required to meet the diagnostic criteria for ARDS and sepsis within 24 h of admission to the ICU. According to the recommendations of the Surviving Sepsis Campaign in 2016 [15] and the extraction method for sepsis-3 patients described by Johnson and coworkers [16], this study included patients suspected of having an infection during ICU admission (within 24 h) with a Sequential Organ Failure Assessment (SOFA) score of ≥2. Clinically suspected infection was diagnosed by bacterial culture positivity and antibiotic administration. According to the Berlin diagnostic criteria of ALI/ARDS, ARDS was defined by the following parameters: (i) mechanical ventilation and positive end-expiratory pressure or continuous positive airway pressure ≥ 5 cmH_2_O; (ii) severe (PaO_2_/FiO_2_ ≤ 100 mmHg), moderate (PaO_2_/FiO_2_ = 100–200 mmHg), or mild (PaO_2_/FiO_2_ = 200–300 mmHg); and (iii) without pleural effusion, lung collapse, lung nodules, or cardiogenic pulmonary edema. Since no cardiogenic pulmonary edema information was available directly from the database, the patients with a pulmonary capillary wedge pressure (PCWP) of ≥18 cmH_2_O were considered to have cardiogenic pulmonary edema.

Patients with the following criteria were excluded: (i) younger than sixteen years, (ii) admitted to the ICU before, (iii) admitted to the cardiothoracic surgery service, (iv) stayed in the ICU for <24 h, (v) suspected with infection > 24 h before and after ICU admission, and (vi) no RDW data available within 24 h of ICU admission or no data on comorbidities.

### 2.3. Data Extraction and Patient Outcomes

The demographic characteristics (e.g., age, sex, and ethnicity), comorbidities (congestive heart failure, anemia, hypertension, chronic respiratory disease, liver disease, and kidney failure) and laboratory data (RDW, platelet count, white blood cell count, blood glucose, urea nitrogen, and serum creatinine), and severity of the disease (SOFA score, ARDS grade, and vasopressin use) of the included patients were extracted from the database. All laboratory parameters were selected for the first measurement. The outcome measure was all-cause MR during the 90 days of ICU admission.

### 2.4. Grouping

Since none of the patients had an RDW less than the normal range (11.5%–14.5%), the patients were categorized into the normal RDW (nRDW) group (RDW ≤ 14.5%) and the increased RDW (iRDW) group (RDW > 14.5%). Locally weighted scatter-plot smoothing (LOWESS) analysis revealed approximately linearly increasing relationship between the RDW and 90- or 30-day all-cause mortalities. Therefore, the iRDW group was subdivided into 2 subgroups using the median value of the RDW as the threshold and then inputting it into the Cox regression model to further explore the impact of an increased RDW on mortality. The resulting three groups were level 1 (RDW ≤ 14.5%), level 2 (14.5% < RDW < 16.2%), and level 3 (RDW ≥ 16.2%).

### 2.5. Treatment of Missing Values

The missing values were <5% for all the variables included in the present study. The normally distributed variables were subjected to mean imputation, while the nonnormally distributed variables were subjected to median imputation. For the categorical variables with missing values, the associated cases were deleted directly.

### 2.6. Statistical Analysis

Categorical variables were analyzed by the chi-square test, and the data are expressed as percentages. Continuous variables were tested with Student's *t* test (normal distribution) or the Mann-Whitney *U* test, and the results are presented as the mean ± standard deviation or median (interquartile range (IQR)). The LOWESS method was employed to assess the general association between RDW and 90- or 30-day all-cause mortalities. The Kaplan-Meier estimator was applied to construct the survival curves of different RDWs, which were compared with the log-rank test. Then, Cox regression was employed to analyze the prognostic factors related to mortality. The variables with *P* < 0.05 in the univariate model were subjected to multivariate Cox regression analysis. Covariate correction was performed using the following models: Model 1 was corrected according to age, sex, and ethnicity; Model 2 = Model 1 + (ARDS grade, comorbidities, and vasopressin use); Model 3 = Model 2 + (RDW, platelet count, and white blood cell count as well as levels of blood sugar, urea nitrogen, and serum creatinine); and Model 4 = Model 3 + (the SOFA scores). Multicollinearity was examined by the variance-inflation factor (VIF), and VIF ≥ 10 (severe multicollinearity) was not allowed in the study.

In the Cox regression model, subgroup analysis was performed according to the severity of illness during ICU admission [17], including the ARDS grade and in combination with septic shock. However, identifying septic shock patients in the aforementioned datasets was difficult because of the lack of access to relevant information. Therefore, it was substituted with vasopressin use within 24 h of ICU admission. Considering that RDW could be affected by the hemoglobin level, another subgroup analysis was carried out according to the association of RDW with anemia. To verify the interaction between the RDW and these variables, the regression model was incorporated with multiplicative interaction terms. The significance level was set at *P* value < 0.05. All statistical tests were conducted with Stata v.16, SPSS v.24, and R v.3.6.3.

## 3. Results

### 3.1. Baseline Characteristics

**Figure 1:**
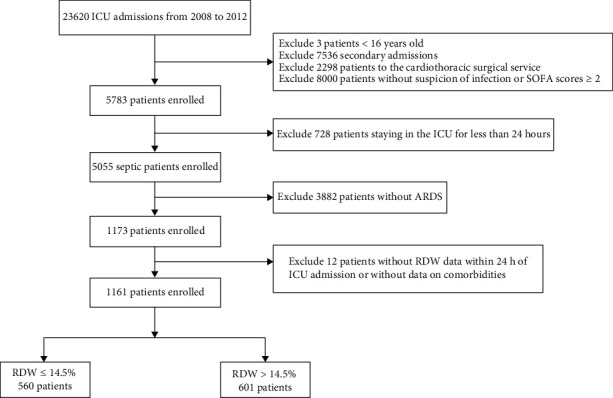
Flow diagram of patient recruitment.

A total of 1161 patients with sepsis-induced ARDS were included in this analysis. The patient selection and data screening processes are illustrated in Figure 1. The overall 90-day all-cause MR was 32.6%. The baseline characteristics of the nRDW and iRDW groups were compared and are presented in Table 1. The overall mean age at ICU admission was 64.2 years, and 56.8% of the patients were male. The frequency of vasopressin use in the iRDW group was remarkably higher than that in the nRDW group (56.6% vs. 48.0%, *P* = 0.004). Moreover, the iRDW group showed a higher proportion of comorbidities, such as congestive heart failure, anemia, high blood pressure, liver disease, and kidney failure. The 90-day MR in the iRDW group was remarkably higher than that in the nRDW group (42.8% vs. 21.8%, *P* < 0.001).

### 3.2. Relationship between RDW and Mortality

**Figure 2:**
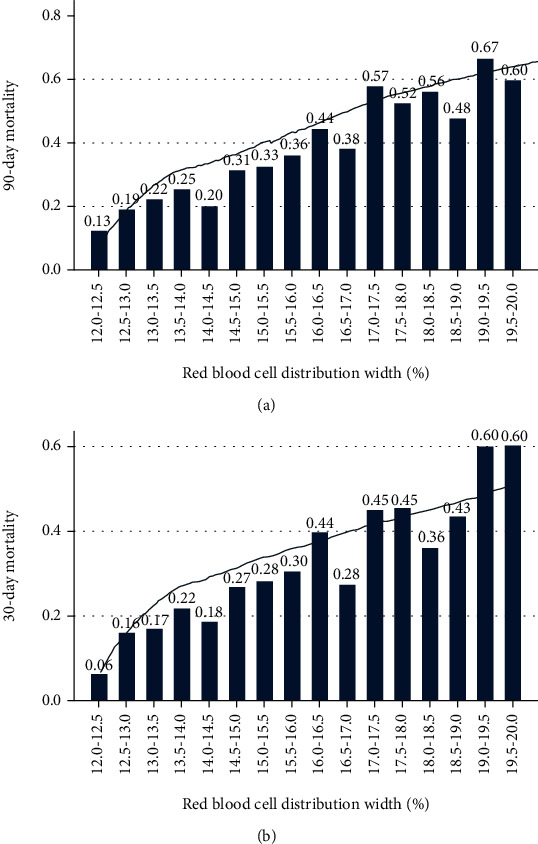
Relationship between RDW and (a) 90-day or (b) 30-day mortality in sepsis-induced ARDS.

**Figure 3:**
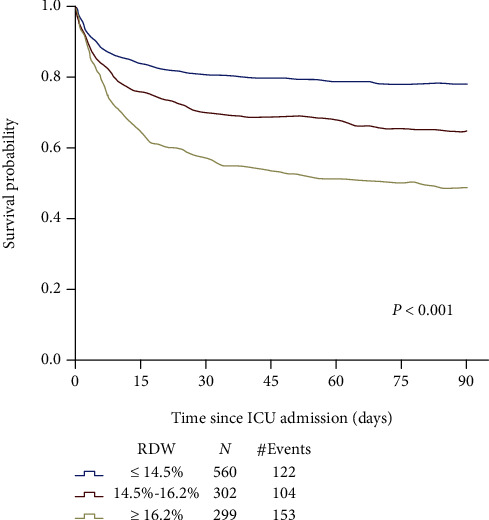
Association between RDW and 90-day overall survival in sepsis-induced ARDS.

An approximately increasing linear relationship was found between RDW and mortality using the LOWESS technique (Figure 2). When the RDW was in the range of 19.0%–19.5%, the 90-day mortality rate was as high as 67%, and the 30-day MR was 60%.  Figure 3 represents the Kaplan-Meier curve describing the association between RDW and 90-day MR in different RDW groups. For various time periods, the level 1 group showed the highest survival rate (*P* < 0.001), followed by the level 2 group.

In the extended multivariate Cox regression model, the level 3 RDW was significantly correlated with the 90-day MR (Table 2). Model 1 showed a hazard ratio (HR) of 2.68 with a 95% confidence interval (CI) of 2.11–3.40. Model 2 had an HR of 2.35 with a 95% CI of 1.83–3.01. Model 3 exhibited an HR of 2.14 with a 95% CI of 1.65–2.78. Model 4 had an HR of 2.07 with a 95% CI of 1.59–2.69. Level 2 exhibited similar results with smaller HR values. Supplementary Table 1 lists the HR values of all covariates in Model 4. When RDW was regarded as a continuous variable, it could also predict 90-day MR (HR, 1.11 per 1% increase; 95% CI, 1.06–1.15) (Table 3).

### 3.3. Subgroup Analysis

**Figure 4:**
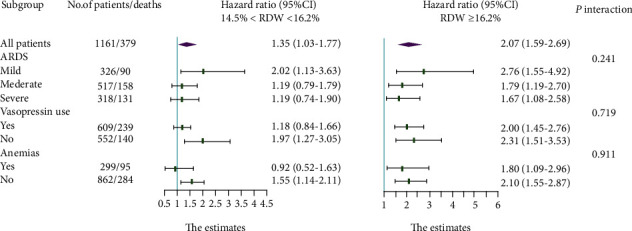
Adjusted hazard ratio in the subgroup analysis.

The results of the subgroup analysis are shown in Figure 4. The *P* values of the interaction between RDW and the degree of ARDS, use of vasopressors, and anemia were 0.241, 0.719, and 0.911, respectively. There were no obvious differences between the RDW and mortality among patients with different degrees of ARDS, whether vasopressin was used and whether anemia was present.

## 4. Discussion

In this study, massive amounts of data were obtained from the MIMIC-III database to assess the prognostic significance of RDW in sepsis-induced ARDS patients. The results demonstrated that RDW and mortality had a roughly linear increasing relationship. Multivariate Cox regression analysis indicated that RDW was independently associated with the high MR of sepsis-induced ARDS patients.

Our results were in good agreement with previous findings that RDW demonstrated good predictive value for many diseases, especially inflammatory diseases. In a retrospective study of 610 patients with severe burns, RDW independently correlated with the occurrence of ARDS. For every 1% increase in RDW, the risk of ARDS was induced by 29% [18]. Ganji et al. conducted a meta-analysis involving 7 studies with 976 patients with pancreatitis. They used the summary receiver operating characteristic (ROC) curve from a bivariate model to predict the prognosis of RDW for patient mortality and obtained an area under the curve (AUC) of 0.757 as well as pooled specificity and sensitivity of 90% (95% CI: 73%–96%) and 67% (95% CI: 51%–80%), respectively [19]. Interestingly, RDW might also be related to the risk of death in the general population. In a study involving 15,852 adults living in the community, the researchers followed up the community residents for 6–12 years and found that the mortality increased twofold from the lowest quintile of the RDW to the highest quintile [20]. Moreover, RDW was also involved in the occurrence of cancers (HR, 1.28; 95% CI, 1.21–1.36), cardiovascular diseases (HR, 1.22; 95% CI, 1.14–1.31), and chronic respiratory diseases (HR, 1.32; 95% CI, 1.17–1.49) [20].

The correlation mechanism between increased RDW and poor prognosis in patients with sepsis-induced ARDS remains elusive. Sepsis-induced ARDS is a systemic inflammatory response syndrome [21]. To date, the link between the inflammatory response and the increase in RDW has been confirmed. Studies have shown a positive correlation between RDW and certain inflammatory biomarkers (erythrocyte sedimentation rate and C-reactive protein) [22, 23], indicating that red blood cell heterogeneity implies the existence of inflammation. Inflammation has adverse effects on bone marrow function, iron metabolism, and red blood cell homeostasis, further leading to the production of a large number of new reticulocytes related to RDW increase [24, 25]. In addition, the increase in oxidative stress boosted RDW by reducing the survival rate of red blood cells and releasing large numbers of premature red blood cells into the circulation [26]. These possible mechanisms might also explain the interaction between RDW and disease severity to a certain extent because the more severe the sepsis-induced ARDS is, the more remarkable the inflammatory response and oxidative stress.

One of the strengths of this research was the large study population, which was sufficient for further stratification and subgroup analysis of RDW. Furthermore, adequate confounding factors were included that might interact with RDW to produce more accurate results because RDW might be affected by a series of factors [27], such as age, sex, anemia, and liver and kidney dysfunction. Nevertheless, this research had some limitations. First, it was not a multicenter retrospective study and hence could have selection bias. Second, only the RDW data within 24 h of ICU admission were analyzed. Thus, follow-up data could be used to verify the findings of this study. Third, identifying patients with septic shock and cardiogenic edema in the datasets was difficult because of the lack of assessments of relevant information; they were replaced with vasopressin use and PCWP value. Last, the MIMIC-III V.1.4 database only included inpatients from 2001 to 2012 but did not include patients from more recent years.

## 5. Conclusion

In summary, this study suggested that RDW is a promising independent prognostic marker of sepsis-induced ARDS and that increased RDW is significantly correlated with poor prognosis. This study provided support for the risk stratification of patients with sepsis-induced ARDS based on RDWs. However, further multicenter prospective research is required to assess the exact mechanism underlying the correlation between RDW and MR and hence further verify the findings.

## Figures and Tables

**Table 1:** Baseline data of the study subjects.

Variable	Total (*n* = 1161)	RDW ≤ 14.5% (*n* = 560)	RDW > 14.5% (*n* = 601)	*P* value
Age, year	64.2 ± 17.3	63.3 ± 17.9	65.0 ± 16.8	0.098
Male, *n* (%)	660 (56.8%)	340 (60.7)	320 (53.3)	0.010
Ethnicity, *n* (%)				0.749
White	811 (69.9)	394 (70.4)	417 (69.4)	
Black	85 (7.3)	43 (7.7)	42 (7.0)	
Other	265 (22.8)	123 (22.0)	142 (23.6)	
SOFA, median (IQR)	7 (4–10)	6 (4–9)	5 (7–11)	<0.001
ARDS stage, *n* (%)				0.018
Mild	326 (28.1)	148 (26.4)	178 (29.6)	
Moderate	517 (44.5)	273 (48.8)	244 (40.6)	
Severe	318 (27.4)	139 (24.8)	179 (29.8)	
Vasopressin use, *n* (%)	609 (52.5)	269 (48.0)	340 (56.6)	0.004
Comorbidities, *n* (%)				
Congestive heart failure	242 (20.8)	98 (17.5)	144 (24.0)	0.007
Chronic pulmonary	291 (25.1)	126 (22.5)	165 (27.5)	0.052
Hypertension	174 (15.0)	55 (9.8)	119 (19.8)	<0.001
Renal failure	198 (17.1)	61 (10.9)	137 (22.8)	<0.001
Liver disease	118 (10.2)	28 (5.0)	90 (15.0)	<0.001
Anemia	299 (25.8)	121 (21.6)	178 (29.6)	0.002
Laboratory data				
White blood cell, 10^9^/L	10.6 (7.6–14.3)	10.9 (8.2–14.3)	10 (6.9–14.3)	0.006
Platelet, 10^9^/L	202 (147–271)	212 (167–278)	186 (125–264)	<0.001
Glucose, mg/dL	133 (107–175)	136 (110–178)	129 (104–173)	0.003
Creatinine, mg/dL	1.1 (0.8–1.7)	1.0 (0.8–1.5)	1.2 (0.8–2.1)	<0.001
Urea nitrogen, mg/dL	22 (15–37)	19.5 (13.0–29.0)	26 (17–44)	<0.001
Clinical outcome				
30-day mortality, *n* (%)	318 (27.4)	104 (18.6)	214 (35.6)	<0.001
90-day mortality, *n* (%)	379 (32.6)	122 (21.8)	257 (42.8)	<0.001

**Table 2:** HR values for the 90-day MR among the 3 RDWs.

	RDW ≤ 14.5%		14.5% < RDW < 16.2%		RDW ≥ 16.2%	
	HR (95% CI)	*P*	HR (95% CI)	*P*	HR (95% CI)	*P*
Model 1	Reference	—	1.61 (1.24–2.10)	<0.001	2.68 (2.11–3.40)	<0.001
Model 2	Reference	—	1.50 (1.15–1.95)	0.003	2.35 (1.83–3.01)	<0.001
Model 3	Reference	—	1.45 (1.11–1.90)	0.006	2.14 (1.65–2.78)	<0.001
Model 4	Reference	—	1.35 (1.03–1.77)	0.028	2.07 (1.59–2.69)	<0.001

**Table 3:** HR values for the 90-day MR with RDW as a continuous variable.

	RDW as a continuous variable (per 1% increase)	
	HR (95% CI)	*P*
Model 1	1.16 (1.47-2.23)	<0.001
Model 2	1.13 (1.09-1.17)	<0.001
Model 3	1.11 (1.07-1.16)	<0.001
Model 4	1.11 (1.06-1.15)	<0.001

## Data Availability

The full dataset used in this study is available from the first author at wanghb53@mail2.sysu.edu.cn. However, reanalysis of the full data for other use requires approval by the MIMIC-III Institute.

## Supplementary Materials

Supplementary Materials Supplementary Table 1: Cox proportional hazards model of factors associated with 90-day mortality among sepsis-induced acute respiratory distress syndrome.
