# Supplementary material for: Prognostic Value of the Red Cell Distribution Width in Patients with Sepsis-Induced Acute Respiratory Distress Syndrome: A Retrospective Cohort Study
Source: Dis Markers. 2021 Jun 2;2021:5543822. doi: 10.1155/2021/5543822 (PMC8189810; doi:10.1155/2021/5543822)
Supplement: Supplementary Materials — Supplementary Table 1: Cox proportional hazards model of factors associated with 90-day mortality among sepsis-induced acute respiratory distress syndrome. [file 5543822.f1.docx]

Supplementary Table 1: Cox proportional hazards model of factors associated with 90-day mortality among sepsis-induced acute respiratory distress syndrome.

| Variables | Univariable analysis | | Multivariable analysis | |
| --- | --- | --- | --- | --- |
|  | HR (95% CI) | P value | HR (95% CI) | P value |
| Age, years |  | **<0.001** |  | **<0.001** |
| <65 years | Reference |  | Reference |  |
| ≥65 years | 1.79 (1.45-2.20) |  | 1.86 (1.47-2.34) |  |
| Sex |  | 0.344 |  |  |
| Female | Reference |  |  |  |
| Male | 0.91 (0.74-1.11) |  |  |  |
| Ethnicity |  | 0.09 |  |  |
| White | Reference |  |  |  |
| Black | 0.75 (0.48-1.17) | 0.204 |  |  |
| Other | 1.21 (0.96-1.53) | 0.107 |  |  |
| SOFA | 1.17 (1.14-1.20) | **<0.001** | 1.15 (1.11-1.19) | **<0.001** |
| ARDS stage |  | **<0.001** |  | 0.228 |
| Mild | Reference |  | Reference |  |
| Moderate | 1.16 (0.89-1.50) | 0.276 | 1.05 (0.81-1.37) | 0.706 |
| Severe | 1.69 (1.30-2.22) | **<0.001** | 1.25 (0.94-1.65) | 0.122 |
| Vasopressin use |  | **<0.001** |  | 0.630 |
| No | Reference |  | Reference |  |
| Yes | 1.77 (1.44-2.18) |  | 0.94 (0.73-1.21) |  |
| Congestive heart failure |  | 0.425 |  |  |
| No | Reference |  |  |  |
| Yes | 1.10 (0.87-1.40) |  |  |  |
| Chronic pulmonary |  | 0.149 |  |  |
| No | Reference |  |  |  |
| Yes | 0.84 (0.66-1.07) |  |  |  |
| Hypertension |  | 0.165 |  |  |
| No | Reference |  |  |  |
| Yes | 1.21 (0.93-1.57) |  |  |  |
| Renal failure |  | **0.003** |  | 0.353 |
| No | Reference |  | Reference |  |
| Yes | 1.44 (1.13-1.83) |  | 0.88 (0.68-1.15) |  |
| Liver disease |  | **<0.001** |  | **0.024** |
| No | Reference |  | Reference |  |
| Yes | 1.86 (1.41-2.46) |  | 1.47 (1.05-2.06) |  |
| Anemias |  | 0.417 |  |  |
| No | Reference |  |  |  |
| Yes | 0.91 (0.72-1.15) |  |  |  |
| WBC, 10^9^/L |  | **<0.001** |  | **<0.001** |
| 4-10 | Reference |  | Reference |  |
| <4 | 2.94 (1.95-4.42) | **<0.001** | 2.31 (1.52-3.52) | **<0.001** |
| >10 | 1.82 (1.46-2.28) | **<0.001** | 1.81 (1.44-2.27) | **<0.001** |
| Platelet, 10^9^/L |  | **<0.001** |  | **0.026** |
| 100-300 | Reference |  | Reference |  |
| <100 | 1.98 (1.50-2.62) | **<0.001** | 0.82 (0.59-1.14) | 0.237 |
| >300 | 1.36 (1.06-1.74) | **0.017** | 1.34 (1.04-1.74) | **0.024** |
| Glucose, mg/dl |  | **0.009** |  | 0.761 |
| 65-110 | Reference |  | Reference |  |
| <65 | 2.21 (1.30-3.75) | **0.003** | 1.17 (0.68-2.00) | 0.572 |
| >110 | 1.01 (0.80-1.28) | 0.913 | 0.97 (0.76-1.22) | 0.777 |
| Creatinine, mg/dl |  | 0.257 |  |  |
| 0.5-1.2 | Reference |  |  |  |
| <0.5 | 1.32 (0.72-2.43) | 0.371 |  |  |
| >1.2 | 1.17 (0.96-1.44) | 0.130 |  |  |
| BUN, mg/dl |  | **<0.001** |  | 0.282 |
| <21 | Reference |  | Reference |  |
| ≥21 | 2.00 (1.61-2.48) |  | 1.14 (0.90-1.46) |  |
| RDW, % |  | **<0.001** |  | **<0.001** |
| ≤14.5 | Reference |  | Reference |  |
| 14.5-16.2 | 1.71 (1.31-2.22) | **<0.001** | 1.35 (1.03-1.77) | **0.028** |
| ≥16.2 | 2.78 (2.19-3.53) | **<0.001** | 2.07 (1.59-2.69) | **<0.001** |

ARDS, acute respiratory distress syndrome; BUN, [blood urea nitrogen](http://www.baidu.com/link?url=J0giB1UdhWWLNXme5D5sVCTtA-w5qCmmQ3msm9bbv81gA-INj38yNwpG90PdoYq9jf97gotsXzs8zapGT2TXua); CI, confidence interval; HR, hazard risk; RDW, red blood cell distribution width; SOFA, Sequential Organ Failure Assessment; WBC, white blood cell.
